# Sub-hourly measurement datasets from 6 real buildings: Energy use and indoor climate

**DOI:** 10.1016/j.dib.2023.109149

**Published:** 2023-04-13

**Authors:** Igor Sartori, Harald Taxt Walnum, Kristian S. Skeie, Laurent Georges, Michael D. Knudsen, Peder Bacher, José Candanedo, Anna-Maria Sigounis, Anand Krishnan Prakash, Marco Pritoni, Jessica Granderson, Shiyu Yang, Man Pun Wan

**Affiliations:** a SINTEF, Department of Architectural Engineering, P.O. Box 124 Blindern, 0314 Oslo, Norway; b NTNU, Department of Energy and Process Engineering, Kolbjørn Hejes vei 1b, 7034 Trondheim, Norway; c Aarhus University, Department of Civil and Architectural Engineering - Building Science,Inge Lehmanns Gade 10, 8000 Aarhus C, Denmark; d DTU Compute, Bygning 324, 2800 Kongens Lyngby, Denmark; e Concordia University, Department of Building, Civil and Environmental Engineering, 1455 De Maisonneuve Blvd. W, Montreal, Quebec, Canada; f CanmetENERGY in Varennes, Energy Efficiency and Technology Sector, 1615 Lionel-Boulet Blvd.,Varennes, QC J3 × 1S6, Canada; g Lawrence Berkeley National Laboratory, Building Technology and Urban Systems, 1 Cyclotron Road, Berkeley, California 94720, United States; h Nanyang Technological University, School of Mechanical and Aerospace Engineering, 639798, Singapore; i Cornell University, Systems Engineering, Ithaca, NY, USA, 14853

**Keywords:** Heating, Ventilation and Air Conditioning (HVAC), High resolution, CSV files, Pseudo-Random Binary Sequence (PRBS), Model identification, Model Predictive Control (MPC), Deep Reinforcement Learning (DRL)

## Abstract

The data presented here were collected independently for 6 real buildings by researchers of different institutions and gathered in the context of the IEA EBC Annex 81 Data-driven Smart Buildings, as a joint effort to compile a diverse range of datasets suitable for advanced control applications of indoor climate and energy use in buildings. The data were acquired by energy meters, both consumption and PV generation, and sensors of technical installation and indoor climate variables, such as temperature, flow rate, relative humidity, CO_2_ level, illuminance. Weather variables were either acquired by local sensors or obtained from a close by meteorological station. The data were collected either during normal operation of the building, with observation periods between 2 weeks and 2 months, or during experiments designed to excite the thermal mass of the building, with observation periods of approximately one week. The data have a time resolution varying between 1 min and 15 min; in some case the highest resolution data are also averaged at larger intervals, up to 30 min.

**Specifications Table**

**Table:** 

Subject:	Architecture
Specific subject area:	Energy use and indoor climate in buildings
Type of data:	Table Image Graph
How the data were acquired:	The data were collected independently for 6 real buildings. The data were acquired by energy meters, both consumption and PV generation, and sensors of technical installation and indoor climate variables, such as temperature, flow rate, relative humidity, CO_2_ level, illuminance. Weather variables, such as temperature, solar radiation, wind speed, were either acquired by local sensors or obtained from a close by meteorological station. Most data are raw, although some variable in some dataset has been filtered, such as the indoor temperature being the average of several sensors placed at different heights or different points. Two experimental designs were used: longer observation periods (2 weeks to 2 months) with normal operation, and shorter observation periods (ca. 1 week) with Pseudo-Random Binary Sequence (PRBS) experiments aimed at exciting the thermal response of the building, when there was no occupation.
Data format:	CSV files, decimal separator '.' Raw Filtered
Description of data collection:	The data were collected with a time resolution from 1 min to 15 min; in some case the highest resolution data are also averaged at larger intervals, up to 30 min.
Data source location:	Dataset 1: Office building in Oslo • Institution: SINTEF • City/Town/Region: Oslo • Country: Norway • Time zone: CET, UTC+1 Dataset 2: ZEB Living Lab • Institution: Norwegian University of Science and Technology (NTNU) • City/Town/Region: Trondheim • Country: Norway • Time zone: CET, UTC+1 Dataset 3: FlexHouse • Institution: Technical University of Denmark (DTU) • City/Town/Region: Roskilde • Country: Denmark • Time zone: CET, UTC+1
	Dataset 4: Varennes Library • Institution: Concordia University • City/Town/Region: Varennes, Quebec • Country: Canada • Time zone: EST, UTC-5 Dataset 5: FLEXLAB • Institution: Lawrence Berkeley National Laboratory • City/Town/Region: Berkeley, California • Country: USA • Time zone: PDT, UTC-7 Dataset 6: Office space at Research Techno Plaza • Institution: Nanyang Technological University (NTU) • City/Town/Region: Singapore • Country: Singapore • Time zone: SGT, UTC+8
Data accessibility:	Repository name: Mendeley Data Data identification number: 10.17632/xztfbtsgys.3 Direct URL to data: https://data.mendeley.com/datasets/xztfbtsgys/3[1].

## Value of the Data

• These data are useful because they offer 6 datasets from different buildings in different countries and climates. The data are of high resolution, from 1 min to 15 min, obtained from precision instrumentation and quality checked by the researchers providing it.
• Both academics and professionals can benefit from these data since they provide the opportunity to develop and test model identification methods on well cured, high quality datasets that were collected by researchers from real buildings, with dedicated measurement equipment.
• These data can be used for identification and validation of controller models for advanced control applications of indoor climate and energy use in building, such as Model Predictive Control (MPC) and Deep Reinforcement Learning (DRL). The data can also be used to calibrate emulators, i.e. detailed physical models, of the building. Where the data have been used for such purposes, reference is given.

## 1 Objective

The data presented here were collected independently for 6 real buildings by researchers of different institutions and gathered in the context of the IEA EBC Annex 81 Data-driven Smart Buildings, as a joint effort to compile a diverse range of datasets suitable for advanced control applications of indoor climate and energy use in buildings. This pool of data becomes now openly accessible to everybody.

## 2 Data Description

### 2.1 Dataset 1: Office Building in Oslo

The building is an old office building built in the 1960s, a concrete construction with brick façade; it has 8 floors plus a basement, with a total floor area of about 3800 m^2^. The basement and 1st floor are laboratories and common areas. 2nd to 7th floor are office areas, a mix of cell-offices and open-plan configurations, while the 8th floor consists of meeting rooms.

The air handling unit (AHU) for the building has a rotating wheel heat recovery unit and an electric reheating element. During normal operation, the air volume flow is controlled by a daily schedule to 0%, 67% or 100%. The supply air temperature by an extract air temperature compensation curve. The air is distributed and extracted to the different floors in one common shaft. In each shaft, the air is split into two branches, covering the northern and southern part of the building, respectively.

Except for the 1st and 8th floor, the room heating in the building is covered by a radiator system supplied by district heating. The district heating substation is located in a nearby building and the heat is transferred to the heating central in the basement. From there it is split into two radiator circuits, supplying the eastern and western façade, respectively. The supply temperatures of the radiator circuits are controlled by an outdoor temperature compensation curve, and the two circuits have individual outdoor temperature sensors. The radiators have no thermostatic control, only manual control valves. This means that the buildings heat supply is only controlled by the outdoor temperature compensation curve, so called weather compensated control (WCC). The list of all variables measured, and their description is given in Table 1.

**Table 1:** Dataset 1 “Office building in Oslo” list of variables and description. The resolution is 5 min, and the period is from 6 to 13 April 2020.

File name	Variable name	Unit of measure	Description
ds1-PRBS-5min	Time	–	Time in Europe/Oslo time in%Y-%m-%d%H:%M:%S UTC offset
	FloRadE	l/s	Volume flow of radiator circuit east
	FloRadW	l/s	Volume flow of radiator circuit west
	TrRadE	C	Return temperature for radiator circuit east
	TsRadE	C	Supply temperature for radiator circuit east
	TrRadW	C	Return temperature for radiator circuit west
	TsRadW	C	Supply temperature for radiator circuit west
	P_radE	W	Calculated thermal power of radiator circuit east
	P_radW	W	Calculated thermal power of radiator circuit west
	P_radTot	W	Calculated total thermal power of radiator circuits
	Tout	C	Outdoor temperature from nearby weather station
	SolGlob	W/m2	Horizontal global radiation from nearby weather station
	WindDir	deg	Wind direction from nearby weather station
	WindSpd	m/s	Wind speed from nearby weather station
	S4_N	C	Temperature of extract air from 4th floor north section
	S4_S	C	Temperature of extract air from 4th floor south section
	S2_A	C	Temperature of extract air from 2nd floor
	S3_S	C	Temperature of extract air from 3rd floor south section
	S6_N	C	Temperature of extract air from 6th floor north section
	S6_S	C	Temperature of extract air from 6th floor north section
	S2–7_A	C	Temperature of extract air from 2nd to 7th floor
	SU-7_A	C	Temperature of extract air from basement to 7th floor
	Tv_sup	C	Ventilation air supply temperature, only available until 2020–04–10 03:00
	Tv_ext	C	Ventilation extract temperature at air handling unit, only available until 2020–04–10 03:00

### 2.2 Dataset 2: ZEB Living Lab

The ZEB (Zero Emission Building) Living Lab is a single-family house with a heated floor area of about 100 m^2^ at NTNU campus in Trondheim. The building has a wooden frame and is highly insulated with 35–40 cm of mineral wool and has a window-to-wall ratio of approximately 20%. The space heating system is flexible, being the building an experimental facility, and can be switched from floor heating to a central radiator to ventilation air. The balanced mechanical ventilation system is equipped with a heat recovery unit and a heating battery; however, this was disabled during these experiments. The building was parted into four zones: two bedrooms, bathroom, and living area.

Two datasets are provided here, one detailed and one minimal, the details of which are given in Table 2. The detailed dataset consists of multiple files, providing different resolution at 1-minute, 5 min and 30-minute intervals. This is available starting from 21 February 2019 19:00 to 13 March 2019 05:30). The minimal dataset has 30-minute averaged values that covers the period from 10 February 2019 12:00 to 13 March 2019 05:30, except for a power failure that disrupted the data collection for approximately three days between the 18th to 21st of February.

**Table 2:** Dataset 2 “ZEB Living Lab” list of variables and description. For the detailed dataset the resolution is 1 min, evt. averaged at larger intervals, and the period is from 21 Feb. to 13 Mar. 2019. For the minimal dataset the resolution is 30 min, and the period is from 10 Feb. to 13 Mar. 2019.

File name	Variable name	Unit of measure	Description
Detailed dataset: ds2-detailed-PT1M ds2-detailed-PT5M ds2-detailed-PT30M	Time	–	The time of the measurement in UTC time
	Tout	C	Outdoor temperature from weather station
	SolGlob	W/m2	Horizontal global radiation from weather station
	SolVerS	W/m2	Vertical total irradiance on south façade (wall)
	WindDir	deg	Wind direction from weather station (roof)
	WindSpd	m/s	Wind speed from weather station (roof)
	PelInt	W	Electric consumption of manikins and appliances
	PelPV	W	PV electricity generation
	CpSp3	%	Circulation pump set point of radiator circuit
	EvRad	%	Electronic valve set point of radiator circuit
	TsRad	C	Supply temperature of radiator circuit
	TrRad	C	Return temperature of radiator circuit
	FloRad	l/h	Actual flow of radiator circuit
	PhRad_actual_power	W	Actual thermal power of radiator circuit
	PhRad_from_energy	W	Calculated thermal power of radiator circuit
	EhRad_energy	kWh	Cumulated energy of radiator circuit
	VolRad	m3	Cumulated volume of radiator circuit
	Tv_sup	C	Ventilation air supply temperature
	Tv_ext_ahu	C	Ventilation extract temperature at AHU
	Tv_ext_kitchen	C	Ventilation extract temperature at kitchen outlet
	Ta_technroom	C	Air temperature of technical room
	RTD29	C	Temperature of living room south, 0.1 m. a. floor
	RTD30	C	Temperature of living room south, 0.8 m.a. floor
	RTD31	C	Temperature of living room south, 1.6 m. a. floor
	RTD32	C	Temperature of living room south, 2.4 m. a. floor
	RTD33	C	Temperature of living room south, 3.2 m. a. floor
	RTD34	C	Temperature of kitchen, 1.6 m. a. floor
	RTD35	C	Temperature of living room south, 0.1 m. a. floor
	RTD36	C	Temperature of living room south, 0.8 m. a. floor
	RTD37	C	Temperature of living room south, 1.6 m. a. floor
	RTD38	C	Temperature of living room south, 2.4 m. a. floor
	RTD39	C	Temperature of living room south, 3.2 m. a. floor
	RTD40	C	Temperature of bedroom west, 1.6 m. a. floor
	RTD41	C	Temperature of bedroom east, 1.6 m. a. floor
	RTD42	C	Temperature of bathroom, 1.6 m. a. floor

Minimal dataset: ds2-minimal-PT30M-including-forecasts	Time	–	The time of the measurement in UTC time
	Tout	C	The outdoor temperature from roof mounted the weather station
	PhRad_actual_power	W	Actual thermal power of radiator circuit
	QestInt	W	Estimated electric consumption of manikins and appliances
	Tv_ext_ahu	C	Ventilation extract temperature at the air handling unit
	Ps	W/m2	Global horizontal solar irradiance from a satellite irradiance service
	Ps_for_1 Ps_for_97	W/m2	Forecasts of solar irradiance for one step ahead, two steps ahead, etc. Retrieved every 30 min with a forecasting horizon of 48 h
	Tout_for_1 Tout_for_97	C	Forecasts of outdoor temperature for one step ahead, two steps ahead, etc. Retrieved every 30 min with a forecasting horizon of 48 h

### 2.3 Dataset 3: Flexhouse

FlexHouse is a part of an experimental facility; the building is heated by remotely controllable electrical heaters, it has temperature logging in each room, and in addition a climate station is located outside the building. The data collected from the five experiments are listed in Table 3, and are all with 5 min resolution. Each room was equipped with wall mounted temperature and room motion sensors, plus Hobo U12–012 sensors were hanged from the ceiling to measure temperature, humidity and light intensity in the center of each room.

**Table 3:** Dataset 3 “FlexHouse” list of variables and description. The resolution is 5 min, and the overall period is from 5 Feb. to 14 Apr. 2009.

File name*	Variable name	Unit of measure	Description
ds3–5MinPRBS1	Time	UTC	The time point of the measurement
ds3–5MinPRBS2	T0, …, T7	C	Temperatures measured with wall mounted sensors
ds3–5MinTHERM1	H0, …, H7	On-Off	Heat control signal for room heaters
ds3–5MinTHERM2	S0, …, S7	Boolean	Room motion sensors
ds3–5MinTHERM3	P	W	Electrical power input to the building, mainly the heaters
	G	kW/m2	Horizontal global radiation from weather station
	Ta	C	Ambient temperature from weather station
	Wd	Degree	Wind direction from weather station
	Ws	m/s	Wind speed from weather station
	HT0, …, HT7	C	Room temperatures measured with free hanging sensors in the center of the rooms
	HRelH0, …, HRelH7	%	Relative humidity measured in center of the rooms
	HI0, …, HI7	Lux	Light intensity measured in center of the rooms
	HCo2_0, …, HCo2_7	Ppm	CO_2_ concentration measured in center of the rooms

⁎ see §0 for a description of the different files.

### 2.4 Dataset 4: Varennes Library

The Varennes Net-Zero Energy library, completed in 2014, is the first net-zero institutional building in Canada. This building is in the town of Varennes, 30 km northeast of Montreal (ASHRAE Climate Zone 6). It is a two-story building, with a floor area of about 2100 m^2^. It comprises a large basement containing the mechanical room and a mezzanine between the two floors. Both floors consist of mainly open plans used primarily as library spaces, with some closed offices on the first floor.

The library has a 110.5 kWp roof-mounted building-integrated photovoltaic (BIPV) array on the south facing part of the roof. From the total BIPV area (706m^2^) about 16% also incorporates thermal recovery from where air is collected and used as pre-heated ventilation air during the heating season. The building's heating and cooling needs are covered by a geothermal system of 8 boreholes feeding four heat pumps with a capacity of 105 kW. Space heating and cooling is distributed by hydronic radiant slabs in the south, east and west perimeter zones. The building is well instrumented, with hundreds of variables recorded in its BAS located in its mechanical room.The list of the available variables and their description is given is Table 4.

**Table 4:** Dataset 4 “Varennes Library” list of variables and description. The resolution is 15 min, and the period is from 1 Jan. to 1 Mar. 2018.

File name*	Variable name	Unit of measure	Description
ds4-Varennes-library	Time	–	time in Canada/Montreal in%y.%d.%Y%H:%M:%S
	hq_imp	kW	Imported power from the grid
	hq_exp	kW	Exported power from the grid
	cons	kW	Total electricity consumption (prod + hq_imp - hq_exp)
	prod	kW	Electricity production from roof PV panels
	net	kW	Net imported power (cons - prod)
	T_slab	C	Average concrete slab temperature
	heating_total	kW	Total heating (thermal) energy
	T_ext	C	Outside temperature
	T_int_setpoint	C	Setpoint for indoor air temperature
	T_int	C	Average indoor air temperature
	T_diff	C	Temperature difference (T_in – T_ext)
	cons_noheating	kW	Plug loads /Electricity without heating loads
	DNI	W/m^2^	Direct normal irradiance
	GHI	W/m^2^	Global horizontal irradiance
	DHI	W/m^2^	Diffused horizontal irradiance
	light_cons	W/m^2^	Electric power used for lighting

### 2.5 Dataset 5: FLEXLAB

FLEXLAB is a well-instrumented experimental test facility at Lawrence Berkeley National Laboratory in Berkeley, California, United States. It has a modular and flexible design with options to modify the building envelope, HVAC and lighting systems, indoor room arrangement etc. It consists of four testbeds, each containing two identical rooms and allowing for side-by-side comparison of a technology against a baseline scenario. The testbeds also have solar panels installed on the roof-top along with multiple Tesla Powerwall batteries. With these technologies, FLEXLAB allows for accurate measurement and evaluation of the behavior of integrated systems (e.g., HVAC and batteries) in providing grid services (e.g., Shift/Shed load when requested) and building services (e.g., visual and thermal comfort).

The data in this test was collected from testbed “XR” that contains two identical and adjacent rooms (Room Cell_A_ and Room Cell_B_), each representing a small commercial office space with a floor area of 57m^2^ and including a large south-facing window. There were three single week-long experiments, conducted to study performance of the advanced control algorithm in optimizing costs incurred for energy efficiency, load shedding and load shifting:

• Energy Efficiency: from 07/09/2020 11:00:00 to 07/17/2020 16:59:59
• Load Shifting: from 07/23/2020 10:00:00 to 07/31/2020 23:59:59
• Load Shedding: fom 08/3/2020 19:00:00 to 08/11/2020 11:00:00

There are four CSV files in the dataset, see Table 5.

**Table 5:** Dataset 5 “FLEXLAB” list of variables and description. The resolution is 1 min, and the period is from 9 Jul. to 11 Aug. 2020.

File name	Variable name	Unit of measure	Description
ds5-external	Time	–	time in America/Los_Angeles in%Y-%m-%d%H:%M:%S
	oat_c	C	Outdoor air temperature
	diffused_irradiance_Wm2	W/m^2^	Diffused Irradiance
	global_irradiance_Wm2	W/m^2^	Global Horizontal Irradiance
	pv_generation_scaled_W	W	PV generation

ds5-cella-state (variable names begin with 'ra') ds5-cellb-state (variable names begin with 'rb')	Time	–	time in America/Los_Angeles time in%Y-%m-%d%H:%M:%S
	ra_plugload_power_W	W	Power consumption by plug loads
	ra_light_power_W	W	Power consumption by lights
	ra_fan_power_W	W	Power consumption by Air Handling Unit supply air fan
	ra_hwp_power_W	W	Power consumption by Hot Water Pump
	ra_chwp_power_W	W	Power consumption by Chilled Water Pump
	ra_hw_th_load_W	W	Power consumption by hot water loop
	ra_chw_th_load_W	W	Power consumption by chilled water loop
	ra_sup_air_temp_C	C	Air Handling Unit supply air temperature
	ra_sup_air_flow_cmh	m^3^/h	Air Handling Unit supply air flow rate
	ra_zone_air_temp1_C	C	Zone Air Temperature from sensor 1. We used the mean zone air temperature for our calculations.
	ra_zone_air_temp2_C	C	Zone Air Temperature from sensor 2
	ra_licor_desk1_1_lux	lux	Indoor illuminance level from desk closest to window on north side
	ra_licor_desk2_1_lux	lux	Indoor illuminance level from desk in middle on north side
	ra_licor_desk3_1_lux	lux	Indoor illuminance level from desk farthest from window on north side
	ra_licor_desk4_1_lux	lux	Indoor illuminance level from desk farthest from window on south side
	ra_licor_desk5_1_lux	lux	Indoor illuminance level from desk in middle on south side
	ra_licor_desk6_1_lux	lux	Indoor illuminance level from desk closest to window on south side
	ra_battery_soc_percentage	%	Cell battery state of charge

ds5-setpoints	Time	–	time in America/Los_Angeles time in%Y-%m-%d%H:%M:%S
	ra_zone_temp_hsp_C	C	Cell_A_ Zone Temperature Heating Setpoint
	ra_zone_temp_csp_C	C	Cell_A_ Zone Temperature Cooling Setpoint
	ra_battery_rate_W	W	Cell_A_ battery charge/discharge rate
	rb_sup_air_flow_sp_cmh	m^3^/h	Cell_B_ Air Handling Unit Supply Air Flow setpoint
	rb_sup_air_temp_sp_C	C	Cell_B_ Air Handling Unit Supply Air Temperature setpoint
	rb_battery_rate_W	W	Cell_B_ battery charge/discharge rate

### 2.6 Dataset 6: Office Space at Research Techno Plaza

Dataset 6 was collected from a testbed office at an academic building, Research Techno Plaza, on the campus of Nanyang Technological University, Singapore. Dataset 6 contains time-series data of variables in the heating, ventilation, and air conditioning (HVAC) system and room space. The data are stored in two CSV files, each containing one month long data: Dec. 2018 – Jan. 2019 for thermostat case (Case1) and Feb. – Mar. 2019 for the MPC Case (Case2). The CSV file “ds6-Case1.csv” contains time series data of HVAC and room air variables when the testbed office was controlled by its original thermostat with a constant set point of 23 °C. The CSV file “ds6-Case2.csv” contains time series data of HVAC and room air variables when the testbed office was controlled by MPC. Differing from the thermostat that tracks a set point temperature of room air, the objective of MPC is to minimize the cooling energy consumed by the HVAC system and minimize the offset between indoor predicted mean vote (PMV) and thermal neutrality (PMV=0) based on the predictive model. In both cases, the room air condition was controlled by a ceiling-mounted variable air volume (VAV) box, which regulated the supply air mass flow rate based the control signals from the thermostat or MPC. Each CSV file contains six variables, as listed in Table 6.

**Table 6:** Dataset 6 “Office space at Research Techno Plaza” list of variables and description. The resolution is 5 min, and the period is from 11 Dec. 2018 to 13 Jan. 2019 for Case1 and from 16 Feb. to 20 Mar. 2019 for Case2.

File name	Variable name	Unit of measure	Description
ds6-Case1	Time	–	Time in Singapore time in d/m/yyyy h:mm
	Supply air temperature	C	Supply air temperature

ds6-Case2	Supply air relative humidity	%	Supply air relative humidity
	Room air temperature	C	Room air temperature
	Room air relative humidity	%	Room air relative humidity
	Supply air mass flow rate	kg/s	Supply air mass flow rate

## 3 Experimental Design, Materials and Methods

### 3.1 Dataset 1: Office Building in Oslo

During the Easter holiday 2020 a PRBS experiment was performed on the unoccupied building in order to generate a rich dataset for model identification. During the experiment:

• The ventilation system was reprogrammed to run constantly at 67%;
• The ventilation supply air temperature was constant at 20 °C;
• The outdoor temperature compensation curves of the radiator shunt valves were fixed to 70 °C;
• The night setback temperature difference was set to −50 °C.

The last two points means that the shunt valves will try to achieve 70 °C during “normal” operation, and 20 °C during “night setback” operation. In this way, the heat supply is turned to maximum when “normal” mode is selected, and turned fully off, when “night setback” mode is selected. The PRBS signal was used to switch between the two modes. During “normal” operation, the actual supply temperature was limited by the available temperature from the district heating. Another important point is that the ventilation air flow rate at 67% is not known.

Thermal power in the two radiator loops was calculated from the measurements of both supply and return temperature, as well as water flow. In order to avoid modifications to the water installations, non-intrusive clamp-on ultrasonic flow meters and Type-T thermocouples were mounted on the pipes’ outer wall and fixed with aluminum tape, before adding insulation on the outside. The flow meters have a specified accuracy of 1.6% of reading ±0.01 m/s, and the Type-T thermocouples have an error specified as maximum of 1.0 °C or 0.75% above 0 °C. All data was logged with a local logger, to avoid issues with wireless data transfer and connection. The same type-T thermocouples were used for air temperature measurements too. The extract air in the ventilation system was measured as a representative temperature for the indoor climate. Sensors and loggers were mounted in the central shaft with temperature sensors connected to selected branches. To place the temperature sensor inside the extract air duct, a hole was bored, and the sensor was attached to a steel wire to ensure location in the middle of the air flow. The temperature was logged with a 5-minute interval.

The data presented here have been used to calibrate a Modelica emulator of the buildings, as described in [2].

### 3.2 Dataset 2: ZEB Living Lab

The dataset has been collected during an experiment that lasted for approximately one winter month, divided into two phases. The first was the excitation phase, which lasted from 10 to 28 February and had the purpose of identifying the thermal dynamics of the building. During this phase a PRBS signal switching the radiator temperature set-point between 21 and 24 °C was created to excite the thermal dynamics of the building. The second phase lasted from 1 to 13 March and had the purpose of investigating the performance of an Economic Model Predictive Control (E-MPC) based on the model identified from the previous phase. Further details on the experimental setup and the E-MPC implementation are available in [3].

Since the ZEB Living Lab is highly insulated, during the experiment the building was heated by a single hydronic radiator. The building was unoccupied but was equipped with four human body sized metal cylinders (dummies) with incandescent lightbulbs to mimic occupants and with other typical household electrical equipment. An air-handling unit (AHU) located in the technical room provided balanced ventilation of approx. 144 m3/h, with a heat recovery effectiveness of approximately 87% as the only pre-heating of the supply air. The hydronic radiator in the living room had a nominal power of 4.7 kW, and the water supply temperature was fixed to approx. 55 °C during the experiment. The water flow was adjusted by a thermostatic radiator valve to track the setpoint determined by either the PRBS signal or the E-MPC logic.

The indoor temperature was represented by the ventilation extract air temperature, which together with the supply air temperature wer measured using PT100 sensors with an accuracy of 0.1Kplaced in the ventilation ducts near the Air Handling Unit (AHU). A Kamstrup heat meter (Multical 602) was used to measure the power delivered to the radiator. Outdoor air temperature was measured by a weather station on the roof of the building while forecasts were obtained from the Norwegian Meteorological Institute weather API. Solar radiation was retrieved from the Solcast web service, providing both real-time data and forecasts based on satellite images. The whole data acquisition system of the ZEB Living Lab is based on the LabVIEW software.

### 3.3 Dataset 3: Flexhouse

The data come from experiments that were designed to estimate the thermal dynamics of the building. Two types of experiments were conducted: one with PRBS signals with high variation of the indoor temperature with the building unoccupied, and one with thermostatic control with and without occupancy in the building. In the two PRBS experiments the heat control signal for room heaters (H0, …, H7) were pre-programmed. In the Thermostatic experiments the set point was constant at 20 °C and the heaters on-off were controlled with a narrow hysteresis band with the temperature feedback sensors being the wall mounted sensors (T0, …, T7). The only variable with missing values is T0.

In total five experiments were carried out in 2009, with the following characteristics:

• PRBS1, 5–11 Feb. A single PRBS signal controls all the heaters in the house, and the house is closed, such that the condition of the house is not changing during the experiment;
• THERM1, 17–26 Feb. Thermostatic control of the heaters, with no activity in the house;
• PRBS2, 27 Feb. – 5 Mar. The house is divided into three separate zones, in which the heaters are controlled with a PRBS signal independently of the other areas;
• THERM2, 6–19 Mar. Thermostatic control of the heaters with activity in the house;
• THERM3, 23 Mar. – 14 Apr, Thermostatic control of the heaters with activity and added thermal mass inside the house.

A detailed description of the experimental design and methods used to acquire the data presented here is available in [4].

### 3.4 Dataset 4: Varennes Library

The data was collected under normal operation of the library for two consecutive months during the heating period (January 2018 -February 2018). For the energy consumption and production, the values are collected in the form of cumulative energy. Data collection internals vary from 5 to 15 min. All the provided data was cleaned and resampled to 15-minute intervals:

• *“hq_imp”* & *“hq_exp”:* These parameters are derived from Hydro-Quebec energy meters. Hydro-Quebec is the largest power utility in Quebec, Canada.
• *“prod”:* Ten 10 kW inverters convert the direct current (DC) output from the PV system to alternating current (AC) which are used by the building or exported to the grid.
• *“cons”:* This is the total energy consumption of the library calculated as *prod + hq_imp – hq_exp*
• *“net”*: The net imported power from Hydro Quebec, *cons – prod*
• *“T_slab”:* This is the average concrete slab temperature. The slab surface is typically between 18 - 29 °C.
• *“*heating_total”: *It includes the heating load supplied by the geothermal heat pumps and an auxiliary electric heater with a capacity of 40* *kW and COP of 1. It does not include energy consumption associated with distribution fans and circulation pumps.*
• *“T_ext”: The outdoor temperature*
• *“T_int_setpoint”:* This parameter indicates the given setpoint profile for the indoor temperature of the library. The library has a different operating schedule for weekdays and the weekend. When occupied the temperature setpoint is maintained between 21 −22 °C and decreases during the night hours to 18 °C.
• *“T_int”:* The air temperature of the zones is averaged based on weighted areas and presented here as a single variable.
• *“T_diff”:* The temperature difference between indoor and outdoor, T_int – T_ext
• *“cons_noheating”:* Plug-loads electricity demand
• *“DNI”, “GHI”, “DHI”:* Solar irradiation data is obtained from the CanmetENERGY-Varennes PV research measurement station located outside of the library.
• *“light_cons”: Electricity demand for lighting*

Further details on the Vareness Library are given in [5].

### 3.5 Dataset 5: Flexlab

Data were collected from two identical rooms in FLEXLAB, namely “Cell_A_” and “Cell_B_”. The configuration of the HVAC system for the two rooms during the experiment was the same. Each room had a single-zone variable-capacity AHU with a single variable-speed fan and two dampers for outdoor air and return air mixing. On the air side of the AHUs sensors measured air volume flow rate, temperature and humidity in each section of the ducts. The water-side of the AHUs was served by a common chiller and boiler. The flow rates and inlet/outlet temperatures in each AHU coil were measured, thus it was possible to calculate the heat flow across the coil. Power consumption of the heating and cooling equipment was estimated from the measurements and the assumption of a fixed efficiency (COP = 3.0 for the chiller and efficiency = 0.95 for the boiler). The only difference between the cells was in the HVAC control logic: a baseline controller was deployed in “Cell_A_”, while a deep reinforcement learning (DRL) based controller was deployed in “Cell_B_” with the goal to optimize the loads and distributed energy resources in the building (see [6] for further details).

Furthermore, each room had six ceiling-mounted light fixtures, six desk stations with desktop computers and other plug-loads, six heat-generating mannequins that emulate occupants, and multiple environmental sensors measuring temperature, indoor light levels, and relative humidity. The energy sources available were a PV system with a capacity of 3.64 kWp a battery storage with a capacity of 7.2 kWh and peak power output of 3.3 kW for each cell. Data were sampled every 10 s or less and then re-sampled to 1 min for analysis.

### 3.6 Dataset 6: Office Space at Research Techno Plaza

The floor area of the testbed office is 46 m^2^ and the floor-to-ceiling height is 3 m. The testbed office is located at level 3 of Research Techno Plaza building on the campus of Nanyang Technological University, Singapore. The testbed office is air-conditioned by a variable air volume (VAV) box, which provides a maximum supply air flow of 1320 m^2^/h. A combined air temperature-relative humidity (RH) sensor was installed in the room space. The same combined air temperature-RH sensor and an air flow rate sensor were installed on the air duct of the HVAC system. The specifications of these sensors are listed in Table 7. The sensor data were recorded by an input/output (I/O) device, which passed the real-time data to a control server. The control server stored the sensor data at 1-min intervals.

**Table 7:** Specifications of the sensors installed in the testbed office room space and HVAC system.

Sensors	Brand	Model	Resolution	Range	Accuracy
Combined air temperature – RH	REGIN	HTDT10–420	0.1 °C	−40 to +60 °C	± 0.01 °C
			0.1% RH	0 to 100% RH	± 2% RH
Air flow rate	SIEMENS	QVM62.1	0.1 m/s	0 to 10 m/s	± 0.2 m/s

The data presented here have been used to identify a controller model, then used in an MPC application, as described in [7].

## Ethics Statements

All datasets were provided with the consent from the building owners, who are the institutions providing the data. Where the building was occupied not exclusively by the building owner, the building has been anonymised.

## CRediT authorship contribution statement

**Igor Sartori:** Conceptualization, Writing – original draft, Writing – review & editing, Data curation. **Harald Taxt Walnum:** Data curation, Investigation, Methodology, Writing – original draft. **Kristian S. Skeie:** Data curation, Investigation, Writing – original draft. **Laurent Georges:** Investigation, Methodology, Writing – original draft. **Michael D. Knudsen:** Data curation, Investigation. **Peder Bacher:** Data curation, Investigation, Methodology, Writing – original draft. **José Candanedo:** Data curation, Investigation, Methodology, Writing – original draft. **Anna-Maria Sigounis:** Data curation, Investigation, Methodology, Writing – original draft. **Anand Krishnan Prakash:** Data curation, Investigation, Methodology, Writing – original draft. **Marco Pritoni:** Supervision, Writing – review & editing. **Jessica Granderson:** Funding acquisition, Supervision. **Shiyu Yang:** Data curation, Investigation, Methodology, Writing – original draft. **Man Pun Wan:** Supervision, Writing – review & editing.

## Declaration of Competing Interest

The authors declare that they have no known competing financial interests or personal relationships that could have appeared to influence the work reported in this paper.

## Data Availability

6_datasets.zip (Original data) (Mendeley Data).
